# Keys to active ageing: new communication technologies and lifelong learning

**DOI:** 10.1186/s40064-016-2434-8

**Published:** 2016-06-17

**Authors:** Mª del Pilar Díaz-López, Remedios López-Liria, José M. Aguilar-Parra, David Padilla-Góngora

**Affiliations:** Research Group Hum-498: Psychological Intervention in Education Development and Guidance, University of Almería, 04120 Almería, Spain; Department of Nursing Science, Physiotherapy and Medicine, Faculty of Education Science, Nursing and Physiotherapy, University of Almería, Crta. Sacramento s/n. La Cañada de San Urbano, 04120 Almería, Spain; Department of Psychology, Faculty of Education Science, Nursing and Physiotherapy, University of Almería, 04120 Almería, Spain

**Keywords:** Adult learning, Elderly, Quality of life, Technological gap, ICT

## Abstract

The purpose of this study is to describe the creation and implementation of an ICT education program for the elderly in various Active Participation Centers in Almería (Spain), assessing its impact on quality of life. From a randomized sample of 200 individuals over the age of 55. Results reveal a high degree of participant satisfaction (76.6 %), as well as improvements in quality of life as compared to the control group after the 3 month program health factor: p = 0.004; leisure and activity factor: p = 0.001; Satisfaction with Life Factor: p < 0.001. The analysis conducted to determine the influence of age and gender on quality of life indicates that there are statistically significant differences in regards to age (the younger groups had higher scores) and gender (the males). This study may serve to facilitate similar works that promotes on-going education in different locations and across the lifespan.

## Background and motivation

Ageing is a natural, on-going, universal, irreversible and heterogeneous phenomenon. It has been a cause of concern since early times (Soldevila et al. 2005). It takes place not only biologically speaking, but also within other areas of life itself, causing changes in affective, social, personal, cultural and economic areas (Huber and Watson 2014).

The low mortality resulting from advances in social and health care services has led to increasing longevity, forcing national and international organizations to reconsider the needs of this group of individuals and to implement strategies and mechanisms that will allow them to age actively and continue to participate in society.

According to estimates from the *United Nations*, 10 % of the global population is over the age of 60, and this is the very age group that is the most affected by the technological gap. There is a major risk of marginalization of the elderly due to their exclusion from the information society, a situation which is exacerbated by the fact that, according to studies, this population is considered incapable of adapting to the new technologies (Querol 2011). An example of this is evidenced by the fact that of the 27 million internet users accounted for by the *Association for the Research of the Media,* only 28.1 % are over the age of 45. In the 16–55 year old age group, 79 % of these individuals have internet access, but in the group of 56–70 year olds, the access decreases to 29 % while in 71–80 year olds, it is only 5 % (Vodafone Fundation 2010).

### Active ageing

In Spain, since the early twenty-first century, there has been an emphasis on economic and social services which have the objective of removing barriers to dependence, disability and mobility. Today, we find ourselves in an era where technical services and communication for the elderly and disabled is essential, in order to promote cohesion and integration of these, the most disadvantaged European citizens, through the acquisition of increased independence, greater degrees of personal autonomy and increased access to knowledge and the understanding of other realities.

The 2012 European Year of Active Ageing and Solidarity between Generations defined active ageing as a way of offering the elderly the possibility of participating fully in society, strengthening their employment possibilities, allowing them to actively contribute through volunteerism, to live independently, adapting housing, infrastructures, technology and transportation. “The fundamental issue is not that we age, but how we do so” (Botella 2005, p. 34). This vision breaks with false myths and prejudices about ageing and, according to the World Health Organization, it permits the elderly to improve their quality of life through their overall health (biological, mental and social), developing their potential and participating in society in accordance with their needs, desires and abilities.

### Lifelong learning among seniors

In order for this experience to be a positive one for the elderly, it should be associated with continuing education, considered the most valuable tool in the processes of social inclusion, active citizenship and personal development, and resulting in an improvement of the quality of life of these adults. It considers the specific needs of each individual, based on their motivations and interests, capabilities and learning rhythms, former education and personal characteristics, also considering the great possibilities that the new technologies of information and communication (ICT) have to offer (Agudo 2006; Agudo et al. 2012; Bermejo 2012; González et al. 2012; Muñoz 2012; Pérez and De-Juanas 2013).

Learning is innate in all humans, a latent process across the life cycle in which skills, experiences, intelligence, memory, and perception, among other qualities, intervene. Continuing education takes on a variety of goals and aims to develop the well-being of the individuals and their quality of life (Bermejo and Miguel 2008; Kallen 1996; Mas-Torelló 2006; OMS 2002; Padilla et al. 2010; Pérez and De-Juanas 2013).

### Social participation and new technology

Social participation is another concept that is currently on the rise. It ensures that individuals interact with others, thereby increasing their self-esteem and sense of belonging. The establishment of appropriate social relationships may play a large role in determining personal success which is associated with happiness and with it, the so-desired quality of life (Hung and Lu 2014; Díaz-López et al. 2013; Padilla et al. 2010; Vicente 2011).

The huge potential of the new technologies in improving quality of life has not gone unnoticed by those working in the field of education, particularly, those working with the elderly. New designs, communication systems, computing, robotics, home automation, product standardization, new technologies applied to technical assistance and many others, make it possible for many elderly individuals to enjoy a more independent life (Abad 2014; Hersh 2014; Padilla and Padilla 2008; Padilla et al. 2010; Pavón 2000).

Today’s technological revolution (computers, tablets, cellular phones, etc.) allows individuals, despite their age and limitations, to have the same rights and opportunities. Education or training should assist older adults to understand the underlying structure of new technologies (Huber and Watson 2014).

It is important that these “digital habits” do not become elements that shall further distance the disabled, elderly and general population. Therefore, day after day, these new technologies are becoming more essential in social and labor integration (Agudo et al. 2012; Hersh 2014; Pavón 2000), the basis of many policies regarding the disabled and elderly in various member countries of European Union.

New technologies are also key in encouraging this social participation, as they allow for users to be in contact with and to communicate with others, to access culture, education, etc. (Agudo et al. 2012; Pavón 2000). Empirical studies on continuous education in the elderly tend to be very limited, particularly those focusing on the ICTs relationship, since there is little research on the variables that may modulate or influence the quality of life of this group (Chen et al. 2008; González et al. 2012; Villar et al. 2010). There is little data about design issues related to technology and older adults (Czaja and Lee 2003; González et al. 2012).

### Research objective

The purpose of this work is to describe the development and implementation of a new information and communication technology (ICT) education program for individuals over the age of 55, held in various Active Participation Centers located in the province of Almería (Andalusia, Spain) and how it affects their quality of life.

## Methods

### Design

The study was conducted based on a pre–post experimental design (with an experimental and control group) using subjects over the age of 55 who attend these Almería active participation centers.

### Research subjects

Randomized sampling was carried out on 200 individuals over the age of 55 in the province of Almería (Spain). It was assigning random numbers to participants (with the website https://www.randomizer.org/). The study was focused on a specific group of seniors who were engaged in Active Participation Centres that offer leisure time activities specifically for seniors in Almería. This segment of senior-oriented social services represents an institutional context for the manifestation of the discourse of active ageing, which declare the aim of enabling seniors to spend their free time actively, adhere to ideas of independence, self-responsibility and activity. These centres offer lectures with a positive vision of ageing based on an active lifestyle, exercise programmes, snack-bar and entertainment for older over 55–60 years or more of age and have retired. The participants, around 1.000 persons in this city are home-based senior citizens who possess normal recognition functions, and can go outside independently.

Finally, the complete information was considered from a total of 112 individuals (experimental group) and 72 in the control group (people who didn’t take a course). There were a total of 62 men and 122 women, between the ages of 57 and 86 years. Table 1 displays the distribution of the participants in the survey in regards to the sociodemographic characteristics that were considered. The exclusion criteria for the study included the failure to sign the informed consent and the failure to systematically attend the program (over 80 % of the sessions).

**Table 1:** Characteristics of experimental and control groups of the study

Variable	Experimental group	Control group
	N	N
Age		
55–60 years	16	10
61–70 years	60	44
71–80 years	28	12
81–86 years	8	6
Total (M; SD)	112 (69.38; 7.04)	72 (67.46; 6.25)
Academic background		
Primary	44	40
Medium	24	10
Professional	20	10
Superior	24	12
Total	112	72
Gender		
Male	32	30
Female	80	42
Total	112	72

### Procedures

Program implementation in the Almería Active Participation Centers was carried out over a 4 month period, from the 18th of November until the 10th of February of 2014, in two weekly sessions of an hour and a half each. Multi-disciplinary professors from the University and undergraduate degree students from the Psychopedagogy Department were responsible for the teaching.

After a meeting held in various centers and the detailed explanation of the program objectives and content, written informed consent was requested from the participants. During the first session, the pre-test questionnaires were completed and the educational proposal was explained to the participants: objectives, content, methodology, educational materials, equipment, participation and communication styles as well as a detailed description of the activities that would form a part of the teaching–learning process, focused mainly on group dynamics (such as games in pairs, role-playing, group reflections, practical activities with cell phones and computers, analysis of diverse points of view, etc.).

This senior educational program was developed in the computer classrooms of the Active Participation Centers. Eighteen sessions of participative and practical work were held. The main objective was for the elderly participants to acquire new skills and abilities in order to optimize their digital competence, improving their emotional state and offering them more autonomy. Specifically, the following objectives were analyzed: “making technological learning fun”; “to handle current technical equipment in order to reduce the gap between the elderly and the young”; “to use computers for basic administrative purposes” and “to overcome the fear that I can’t”. The teaching conducted by the Psychopedagogy students included the exchange of intergenerational experiences.

### Session programming

An initial block of activities focused on content that was related to mobile telephones, androids and iPhones, etc. Activities were carried out in small groups, pairs and individuals, whereby participants had to, for example, take a photograph and send it via Bluetooth to a colleague. They also learned to use applications in order to contact and communicate with others free of charge.

The second block consisted of the use of credit cards, bank operations and purchases over the Internet. Practical simulations were carried out for these, in order to help the participants overcome their fear of the same. And the final block was dedicated to computer-related activities. First, PC usage and handling sessions were carried out: file organization, the use of word processing, etc. and then, Internet navigation was covered: electronic banking, weather consultation, the news, requesting a doctor’s appointment, as well as use of applications and programs such as Skype or videoconferences.

### Research tools

To evaluate the repercussions of the ICTs education program on the quality of life of the elderly, the Brief Questionnaire on Quality of Life was used (CUBRECAVI, Fernández-Ballesteros and Zamarrón 2007). This tool is based on a multi-dimensional vision of quality of life, consisting of 19 items grouped in nine domains that reference health, social integration, functional skills, leisure and activities, environmental quality, life satisfaction, education, income, social and healthcare services as well as quality of life in general. Internal consistency and reliability ranged from 0.92 to 0.70. Cronbach’s alpha was calculated in CUBRECAVI domains with 2 or more items: Health (0.70), Social Integration (0.71), Leisure and Activity (0.74), Functional Skills (0.92) and Environmental Quality (0.78).

It can be self-administered or applied by interview, requires primary educational level and takes about 15–20 min. Scores in each domain have a range of 1–4, and there is no QoL total score, but a profile with all the QoL ingredient. Norms in percentiles for people living in the community for all QoL domains with a standard sample.

The elderly participants in the experimental group also completed an ad hoc questionnaire on satisfaction with the ICTs education program, to determine if their expectations were met, if benefits were obtained, if their interest was held, if the experiences, activities and materials were appropriate, if they were satisfied with the teachers, group, etc.

### Data analysis

Data analysis was conducted using the SPSS version 22.0 statistics package, revealing the descriptive results from the pretest and posttest of the experimental and control groups. Subsequently, the means of the posttests were compared with those of the pretests for each group, using the Wilcoxon test for related samples. To determine the existence of differences between groups, the U Mann–Whitney test was used for independent samples. Third, *r* was used to assess the magnitude of the change produced by the intervention (effect size). Similarly, the percentage between posttest and pretest scores for both groups was calculated. Finally, a analysis with U Mann–Whitney test and Kruskal–Wallis was conducted to assess the influence of gender and age complemented by the effect size using *r* and *Cramer´ V*.

## Results

Table 2 presents the means and standard deviations of the study variables derived from the CUBRECAVI corresponding to the experimental and control groups for each study phase. The control group did not reveal any differences in the quality of life means that were assessed prior to the first assessment and after the 3 month period. On the other hand, in the experimental group, we find that changes occurred in all of the factors making up quality of life after the completion of the educational program period.

**Table 2:** Quality of life pretest and posttest for experimental and control groups

Quality of life variables (CUBRECAVI) Range of 1–4	Experimental group					Control group				
	Pretest n = 112	Posttest n = 112	Pre-post			Pretest n = 72	Posttest n = 72	Pre-post		
	M (SD)	M (SD)	*Z*	*p*	*r*	M (SD)	M (SD)	*Z*	*p*	*r*
Subjective health	2.79 (0.65)	3.11 (0.71)	−2.56	0.011*	−0.241	2.46 (0.89)	2.43 (0.85)	−1.00	0.317	−0.117
Objective health	3.20 (0.49)	3.42 (0.39)	−2.77	0.006**	−0.262	3.22 (0.52)	3.20 (0.51)	−1.16	0.244	−0.136
Mental health	2.90 (0.64)	3.11 (0.50)	−2.53	0.011*	−0.239	2.94 (0.56)	2.87 (0.58)	−1.16	0.246	−0.136
Health factor	2.97 (0.50)	3.21 (0.43)	−2.69	0.007**	−0.254	2.89 (0.58)	2.86 (0.56)	−1.96	0.051	−0.231
Social integration factor	3.10 (0.52)	3.25 (0.55)	−2.02	0.044*	−0.190	3.18 (0.66)	3.16 (0.70)	−0.53	0.599	−0.062
Functional skills factor	3.17 (0.89)	3.33 (0.75)	−2.49	0.013*	−0.235	3.23 (0.63)	3.21 (0.62)	−0.28	0.777	−0.033
Leisure and activity factor	2.54 (0.41)	2.69 (0.43)	−2.68	0.007**	−0.253	2.36 (0.37)	2.33 (0.36)	−0.51	0.610	−0.060
Environmental quality factor	2.88 (0.22)	2.94 (0.08)	−2.64	0.008**	−0.249	2.95 (0.10)	2.93 (0.10)	−1.83	0.067	−0.215
Satisfaction with life factor	3.14 (0.77)	3.58 (0.56)	−3.44	0.000***	−0.325	3.00 (0.71)	2.91 (0.69)	−1.34	0.180	−0.158

Wilcoxon test for related samples

* *p* ≤ 0.05; ** *p* ≤ 0.01; *** *p* ≤ 0.001

Table 3 reveals that there were no differences between the initial assessment of the elderly participants’ quality of life in the experimental group and the control group. However, after the 3 month period, statistical differences were found between both groups for most of the analyzed variables. Subjective health, the health factor, activity and leisure, as well as satisfaction with life were higher in the experimental group participants as opposed to those from the control group. As seen from the effect size, with *r* was found to be moderate.

**Table 3:** Differences between groups in the Pretest and Posttest

Variable (CUBRECAVI)	Pretest experimental (n = 112) and control (n = 72) groups				Posttest experimental (n = 112) and control (n = 72) groups			
	*U*	*W*	*p*	*r*	*U*	*W*	*p*	*r*
Subjective health	784.00	1345.00	0.236	−0.087	488.00	953.00	0.001***	−0.252
Objective health	816.00	2301.00	0.511	−0.048	670.00	1166.00	0.100	−0.121
Mental health	826.50	2422.50	0.288	−0.078	796.00	1391.00	0.187	−0.097
Health factor	802.50	1298.50	0.860	−0.012	504.50	969.50	0.004**	−0.210
Social integration factor	569.00	1844.00	0.528	−0.046	515.00	746.00	0.899	−0.009
Functional skills factor	868.50	1498.50	0.716	−0.026	818.00	1484.00	0.156	−0.104
Leisure and activity factor	567.50	1032.50	0.192	−0.096	362.00	797.00	0.001***	−0.245
Environmental quality factor	703.00	2299.00	0.069	−0.173	940.00	1535.00	0.906	−0.008
Satisfaction with life factor	900	1566.00	0.354	−0.068	948.00	1164.00	0.000***	−0.330

U Mann–Whitney test for independent samples

* *p* ≤ 0.05; ** *p* ≤ 0.01; *** *p* ≤ 0.001

The analysis conducted to assess the influence of age and gender on quality of life (Table 4) indicates that there are statistically significant differences in regards to age (the younger groups had higher scores). On the other hand, there were significant differences found based on gender, that is, men had a higher mean than women. However, based on the effect size reflected via *r,* these differences were found to be moderate.

**Table 4:** Variables between which significant differences are found based on gender using U Mann–Whitney test and age using Kruskal–Wallis

Variables	Gender	*M*	*p*	*U*	W	*r*
	Male (*n* = 32) Female (*n* = 80)					
Subjective health pretest	Male	3.58	0.000***	131.50	911.50	−0.353
	Female	2.61				
Objective health pretest	Male	3.51	0.000***	112.50	853.50	−0.343
	Female	3.24				
Mental health pretest	Male	3.12	0.004**	163.00	983.00	−0.271
	Female	2.95				
Mental health postest	Male	3.03	0.004**	161.50	981.50	−0.275
	Female	2.84				
Health factor pretest	Male	3.40	0.000***	87.00	790.00	−0.383
	Female	2.94				
Social integration factor pretest	Male	3.37	0.001***	86.00	789.00	−0.324
	Female	2.84				
Functional skills factor	Male	3.77	0.016*	169.50	835.50	−0.228
	Female	3.10				

	Years	*M*	*p*	*X* ^2^	*Gl.*	*V*
Subjective health postest	51–60 years (n = 16)	3.35	0.008**	11.72	3	0.265
	61–70 years (n = 60)	2.63				
	71–80 years (n = 28)	2.58				
	+ de 81 years (n = 8)	3.25				
Objective health pretest	51–60 years (n = 16)	3.46	0.024*	9.40	3	0.237
	61–70 years (n = 60)	3.27				
	71–80 years (n = 28)	3.24				
	+ de 81 years (n = 8)	3.54				
Objective health postest	51–60 years (n = 16)	3.65	0.001***	15.50	3	0.303
	61–70 years (n = 60)	3.20				
	71–80 years (n = 28)	3.16				
	+ de 81 years (n = 8)	3.55				
Mental health pretest	51–60 years (n = 16)	3.31	0.037*	8.48	3	0.223
	61–70 years (n = 60)	3.25				
	71–80 years (n = 28)	2.72				
	+ de 81 years (n = 8)	2.87				
Social integration factor postest	51–60 years (n = 16)	2.62	0.007**	12.26	3	0.273
	61–70 years (n = 60)	3.45				
	71–80 years (n = 28)	2.93				
	+ de 81 years (n = 8)	3.37				
Functional skills factor postest	51–60 years (n = 16)	3.93	0.039*	8.39	3	0.221
	61–70 years (n = 60)	3.49				
	71–80 years (n = 28)	2.86				
	+ de 81 years (n = 8)	3.46				

* *p* ≤ 0.05; ** *p* ≤ 0.01; *** *p* ≤ 0.001

Regarding the analysis of the satisfaction questionnaire on the ICTs education program, Table 5 reveals that participants were very satisfied with this program and were willing to increase their knowledge by participating in future editions.

**Table 5:** Percentage of satisfaction of individuals from the experimental group in regards to the ICT educational program

Questions	Very satisfied	Somewhat satisfied	Slightly satisfied	Not satisfied
Have you obtained benefits for your daily life from the information received in the ICT education program?	60.7 % n = 68	37.3 % n = 42	0 n = 0	2 % n = 2
Did the program content meet your expectations?	57.2 % n = 64	37.3 % n = 42	5.5 % n = 6	0 n = 0
Were the presentations and orientations presented by the teacher appropriate for your understanding of the topics?	74.5 % n = 84	25.5 % n = 28	0 n = 0	0 n = 0
Did participating in the class increase your discussion, motivation and interest?	43 % n = 48	57 % n = 64	0 n = 0	0 n = 0
Did the teacher help you to understand the content of the classes?	72.2 % n = 81	25.8 % n = 29	2 % n = 2	0 n = 0
Are you satisfied with the teacher’s role in the classes?	82 % n = 92	18 % n = 20	0 n = 0	0 n = 0
Do you believe that the experiences shared with the group are important?	60.7 % n = 66	37.3 % n = 42	2 % n = 2	0 n = 0
Are you satisfied with the activities conducted in the group and what they provided?	60.7 % n = 67	39.3 % n = 45	0 n = 0	0 n = 0
Were the materials used (documents, computers, videos) helpful and appropriate for the program?	46.4 % n = 52	49.1 % n = 55	4.5 % n = 5	0 n = 0
Would you like to participate in another ICT program in the future?	100 % n = 112	0 n = 0	0 n = 0	0 n = 0
ICT education program score (1 lowest score, 5 highest/most positive)	76.6 %/5 points n = 86		23.4 %/4 points n = 26	

## Discussion and conclusions

Based on these analyses, we conclude that the experimental group had a higher mean for the quality of life questionnaire posttest with respect to the pretest, as compared to the control group. The benefits occurring in the individuals participating in the educational program as compared to those from the control group, appeared mainly in the areas of subjective health and life satisfaction. This information supports findings from other studies that suggest that the health factors is one of the most important within the satisfaction and quality of life of the elderly individuals, since they tend to be mainly concerned with their health, questioning of their experiences and personal satisfaction, with all of this affecting their emotional well-being (Gallegos-Carrillo et al. 2006; Rodríguez-Marín et al. 1993; Uribe, Valderrama and Molina 2007). Another recent study shows that social capital does not directly predict computer self-efficacy but depends on learning satisfaction as a mediator (Kuo et al. 2013). ICT utilization plays an important role in the well-being of the older femeles who often are unable to use ICT regularly due to their cultural roles.

Similar studies have been carried out in Latin American countries such as Argentina and Mexico. The Rio Cuarto National University has found clear benefits of programs and workshops held for the elderly, since they have expressed a great satisfaction with their participation, highlighting the benefits received from the continuous education (Boarini et al. 2006). They offer testimonials such as the following: “It is gratifying to be able to use technologies without this being taboo for people who have never had contact with them”; “The workshop gave us what we were looking for, entering a new world and one without limits” revealing how these digital competence programs for the elderly help to promote their social integration and improve their quality of life (Boarini et al. 2006; González et al. 2012).

Workshops on basic computer and Internet education for the elderly in the community of San Cristóbal Ecatepec (Mexico) revealed that working with this population may offer a new vision of more active individuals who are capable of facing the challenge of using ICT; rejecting the idea of passivity, hopelessness, deterioration, etc. (Aldana et al. 2012; Mendoza et al. 2005; Villar et al. 2010).

In Spain, a recent study conducted at Senior Citizen’s Centers in Cuenca on ICTs activity in the elderly, revealed relationships between implication in the activities, experience with computers, and belief in the usefulness of learning new technologies. The elderly also indicated that this education helped them to maintain their mental activity, believing it to be a means of social participation and lifelong learning (González et al. 2012).

When considering the age variable, results have revealed that the younger participants in the study perceived greater health benefits, as indicated previously in a study by Barrios et al. (2003) or they were more likely to volunteer in these types of activities (González et al. 2012). In fact, age and disability barriers may be partially removed with the appropriate use of new technologies and technical assistance, however, it is first necessary to break down the mental barriers which tend to limit these individuals. In an article by Pavón (2000, p. 134), an interview with an elderly participant, Maria, 71 years of age, put it perfectly: “I am hesitant to touch things (referring to the functions of the television, video, etc.) because they scare me. When my grandchildren visit, they tell me what these things are for, but I tell them to leave them alone, because I am afraid that they will break them”. A study by González et al. (2012) revealed that the elderly with less computer experience believe that the classes would be more complicated; and the most involved participants feel that these classes would be useful in their everyday lives.

It is important to note that one of the difficulties encountered during this study was the fact that its subject group does not tend to participate in the continuous workshops or education programs offered by the Active Participation Centers or Universities for seniors. Therefore, some of the greatest challenges of this educational program study held in Almería, consisted of ensuring participant attendance, adjusting the difficulty level of the proposed activities, carrying out participatory works that were related to their motivations and interests and promoting a sense of belonging, care, respect, inclusion, personal growth, etc. in the group. And we should not overlook the fear produced when openly exposing one’s emotions, -the fear of the unknown, of breaking routines, etc. Therefore, it was essential to decipher their signs, use their language, understand their ways of life and interpret their cultural habits. This meant that the group was ever more cohesive and eventually, all of the participants were implicated. This reveals the need for the appropriate training to ensure that those leading these courses have specific knowledge, including a full understanding of the reality of the student group. Self-restraint, the ability to listen, patience, respect, attention to diversity -all of these are essential when educating the elderly (Luque 2004; Villar et al. 2010).

This program reveals the usefulness of these ongoing ICTs education programs for the elderly. An attempt has been made to thoroughly explain its development and implementation processes in Almería, so that it may be transmitted to the scientific community and replicated in other communities. Additional research needs to be carried out in this area, in order to determine its repercussions in the elderly, offering strengths and weaknesses existing in the top programs that are directed to this population. Another limitation encountered is the difficulty of conducting these studies in additional active participation centers so as to attain larger sample sizes. Unfortunately, limited resources are devoted to these activities. In the future, it will be necessary to advance this work by including additional centers and other Spanish provinces, as well as the use of the regular center educators to lead the training.

To ensure the participation of the elderly, a new mentality in educational and social actions is needed, in order to connect them to the new technologies, ensuring that these technologies are appropriate for their needs, expectations and interests, wherever they may be located (Active Participation Centers, associations, etc.), and not only in university courses for the elderly (Villar et al. 2010) In this way, society’s goal of achieving equality and full participation of the elderly may become a reality.

This study aims to promote new works that will extend the research on ongoing education across the lifespan and that will promote active ageing and access to new technologies by the elderly population, a population that is currently at risk for social exclusion.

## Abbreviations

ICT: technologies of information and communication
QoL: quality of life
